# Antifungal Activity of a Hydroethanolic Extract From *Astronium urundeuva* Leaves Against *Candida albicans* and *Candida glabrata*

**DOI:** 10.3389/fmicb.2019.02642

**Published:** 2019-11-15

**Authors:** Bruna Vidal Bonifácio, Taissa Vieira Machado Vila, Isadora Fantacini Masiero, Patrícia Bento da Silva, Isabel Cristiane da Silva, Érica de Oliveira Lopes, Matheus Aparecido dos Santos Ramos, Leonardo Perez de Souza, Wagner Vilegas, Fernando Rogério Pavan, Marlus Chorilli, José Luis Lopez-Ribot, Taís Maria Bauab

**Affiliations:** ^1^School of Pharmaceutical Sciences, São Paulo State University (UNESP), Araraquara, Brazil; ^2^Department of Biology and South Texas Center for Emerging Infectious Diseases, The University of Texas at San Antonio (UTSA), San Antonio, TX, United States; ^3^Institute of Chemistry, São Paulo State University (UNESP), Araraquara, Brazil; ^4^Institute of Biosciences, São Paulo State University (UNESP), São Vicente, Brazil

**Keywords:** *Astronium urundeuva*, *Candida* sp. biofilm, checkerboard, resistance, vulvovaginal candidiasis

## Abstract

We have previously reported on the activity of different extracts from *Astronium* sp. against *Candida albicans*, with the hydroethanolic extract prepared from leaves of *A. urundeuva*, an arboreal species widely distributed in arid environments of South America and often used in folk medicine, displaying the highest *in vitro* activity. Here we have further evaluated the antifungal activity of this extract against strains of *C. albicans* and *C. glabrata*, the two most common etiological agents of candidiasis. The extract was tested alone and loaded into a nanostructured lipid system (10% oil phase, 10% surfactant and 80% aqueous phase, 0.5% Poloxamer 407^®^). *In vitro* susceptibility assays demonstrated the antifungal activity of the free extract and the microemulsion against both *Candida* species, with increased activity against *C. glabrata*, including collection strains and clinical isolates displaying different levels of resistance against the most common clinically used antifungal drugs. Checkerboard results showed synergism when the free extract was combined with amphotericin B against *C. albicans*. Serial passage experiments confirmed development of resistance to fluconazole but not to the free extract upon prolonged exposure. Although preformed biofilms were intrinsically resistant to treatment with the extract, it was able to inhibit biofilm formation by *C. albicans* at concentrations comparable to those inhibiting planktonic growth. Cytotoxicity assays in different cell lines as well as an alternative model using *Artemia salina* L. confirmed a good safety profile of the both free and loaded extracts, and an *in vivo* assay demonstrated the efficacy of the free and loaded extracts when used topically in a rat model of vaginal candidiasis. Overall, these results reveal the promise of the *A. urundeuva* leaves extract to be further investigated and developed as an antifungal.

## Introduction

There are approximately 200 species in the genus *Candida*, several of which can inhabit several environments in the body such as the oral cavity, oropharynx, bronchial secretions, skin folds, feces, urine and vagina (Achkar and Fries, 2010; Khan et al., 2018; Qadir and Asif, 2019). Some species are limited to certain body regions, but others, including *C. albicans*, the most common cause of candidiasis associated with approximately 50% of cases, are widely distributed. Warmer and more humid places, such as the oral and the vaginal cavity, are considered most favorable for the development of the infection. For example, vulvovaginal candidiasis (VVC) is considered the most common infection affecting around 75% of women at least once in their lifetime, which could also present frequent episodes of recurrence (Santos Ramos et al., 2015). Among the non-*albicans Candida* species, *C. glabrata* is considered the second most frequent causative agent of candidiasis (Gabaldón and Carreté, 2016; Whaley et al., 2017). Some infections are also caused by more than one *Candida* species, especially oral candidiasis, which may involve both *C. albicans* and *C. glabrata* (Nakamura-Vasconcelos et al., 2017). Very often these infections, including mucosal and invasive candidiasis, are associated with biofilm formation, with biofilms serving as a protective environment against external insults (Uppuluri et al., 2009).

The current antifungal arsenal is mostly limited to amphotericin B, azoles and echinocandins (Pierce et al., 2013). The high incidence of the different forms of candidiasis in an increasing number of compromised patients and the development of resistance against these conventional antifungal agents point to the urgent need to discover and develop novel therapeutics against infections caused by *Candida* spp. (Ngo et al., 2016; Perlin et al., 2017). Natural products have historically represented the main starting point of compounds for therapeutic use and a majority of antibiotics have been traditionally obtained from natural sources. Several plants have shown important *in vitro* and *in vivo* antimicrobial activities, justifying even more the intense search for traditional medicine focused on antimicrobial characterization of plants. The biological activity of medicinal plants from different regions of the world has been studied by several groups of researchers based on tradition. A number of studies have reported the presence of various substances, including extracts and vegetable oils which are effective to control the growth of a wide variety of micro-organisms including fungi (Swamy et al., 2016; Rajkowska et al., 2017).

*Astronium* (Anacardiaceae) is a genus containing several medicinal species, including *Astronium urundeuva* (Allemão) Engl., commonly known as aroeira-preta or aroeira-do-sertão. This is an arboreal species widely distributed in South America, especially in the arid environment of Cerrado, pollinated by wind and containing a small flower. The size of this specie varies according to region of occurrence, but usually features 30 m high, with inflorescence between July and September and ripening of the fruit from September to October, consisting of a single seed (Machado and Oliveira, 2014; Leite et al., 2017). It is considered one of the most popular traditional medicinal plants in Northeastern Brazil as treatment to various diseases, including gastritis, gastric ulcers, cervicitis, vaginitis, hemorrhoids and as anti-inflammatory and natural healing (Calou et al., 2014; Machado and Oliveira, 2014; Resende et al., 2015). Previous studies reported the major components of *Astronium* sp. extracts, including chalcones, flavonoids precursors, essential oils, hydrolizable and condensed tannins that are considered to be the main responsible for the therapeutic activities. The presence of volatile oils justifies the typical aroma found in *A. urundeuva* leaves (Costa et al., 2014; Machado et al., 2016; Leite et al., 2017). We have previously reported on the *in vitro* antifungal activity of different extracts from a number of *Astronium* sp., with the hydroethanolic plant extract prepared from leaves of *A. urundeuva* displaying the most potent activity against *Candida albicans* (Bonifácio et al., 2015). Moreover this extract, both free and loaded into a nanostructured lipid system, displayed activity in an *in vivo* model of vulvovaginal candidiasis using Wistar rats (Bonifácio et al., 2015).

Here we have further evaluated the antifungal activity of this extract against different strains of *C. albicans* and *C. glabrata*, the two most common etiological agents of candidiasis. *In vitro* and *in vivo* results indicate the anticandidal activity and properties of both the free extract and an improved microemulsion formulation, and confirm the promise of the *A. urundeuva* leaves extract to be further developed as an antifungal for the treatment of candidiasis.

## Materials and Methods

### Preparation of and Hydroethanolic Extract of *A. urundeuva* Leaves

*A. urundeuva* leaves were collected in Bálsamo, São Paulo, Brazil, in November 2007 and authenticated in Campinas by Prof. Dr. Jorge Tamashiro (Institute of Biosciences, State University of Campinas – UNICAMP). A voucher specimen of this plant was deposited at the Herbarium of the Institute – UNICAMP and identified as SANO N° 1446. The hydroethanolic extract of *A. urundeuva* was obtained by exhaustive percolation method as previously described (Simões et al., 2017), and then, a rotary evaporator was used under reduced pressure (50°C). After being transferred to a tared glass, the extract was maintained in the fume hood until it was completely dried and was freeze-dried when necessary. For use in susceptibility experiments the free extract was prepared by solubilizing the crude extract in DMSO and miliQ water (at a ratio of 1:9) at a final concentration of 2000 μg/mL.

### Preparation of a Loaded Extract

The loaded extract was prepared by loading the crude extract into a nanosystem composed of cholesterol (10%) as an oil phase, phosphate buffer saline (PBS, pH 7.4) as an aqueous phase (80%), and a surfactant mixture of soybean phosphatidylcholine (SPC)/polyoxyethylene (20) cetyl ether (Brij^®^58) (10%) as previously described (Bonifácio et al., 2015), with 0.5% of Poloxamer 407^®^ added in order to promote better bioadhesion characteristics (for *in vivo* testing). The formulation was obtained by sonication in a rod sonicator (Q700 – Qsonica^®^ 700 W) with an ice bath. Briefly, the mixture was sonicated for 10 min with an interval of 30 s every 2 min. After preparing the microemulsion, the extract was added to the formulation and then the sonication process was performed once again but this time for only 3 min in continuous mode.

### *In vitro* Susceptibility Testing to Examine the Antifungal Activity Against Collection of Strains and Clinical Isolates of *C. albicans* and *C. glabrata*

We evaluated the activity of the free and loaded extracts against *C. albicans* ATCC 10231 and SC5314 strains, and against *C. glabrata* ATCC 2001 strain. In addition the free extract was also tested against some *C. albicans* strains that have mechanisms of resistance that have been previously characterized in the Lopez-Ribot laboratory (Perea et al., 2001; Li et al., 2004; Vila et al., 2015). We also used several *C. albicans* clinical isolates with previously characterized molecular mechanisms of azole resistance, TW2, TW3 and TW17, which represent a series of matched susceptible and resistant isolates from the same patient with oropharyngeal candidiasis (White, 1997). In addition, we used a set of genetically engineered *C. albicans* “gain of function” strains in key transcriptional regulators of azole resistance including *TAC1*, *MRR1* and *UPC2* (a kind gift from David Rogers) (Schubert et al., 2011; Flowers et al., 2012). In the case of *C. glabrata*, we also used a series of clinical isolates with different levels of resistance against azoles or echinocandins, which were provided by the Fungus Testing Laboratory (FTL) at The University of Texas Health Science Center at San Antonio. Antifungal susceptibility testing was performed using a broth microdilution technique following the methodology described in document M27-A3 published by the Clinical and Laboratory Standards Institute (CLSI) (Clinical and Laboratory Standards Institute, 2008) with minor modifications, with determination of MIC_90_ as the concentration that inhibits 90% of the fungal yeast growth when compared to the growth control. MIC values were initially evaluated by visual analysis and confirmed by spectrophotometry at 490 nm in a microtiter plate reader.

### Determination of Minimum Fungicidal Concentration (MFC)

In order to verify that the extract was able to kill the yeast cells (fungicidal effect) the plates were also evaluated for MFC. Briefly, aliquots from each well from susceptibility testing assays were transferred to plates containing Sabouraud (SDA), which were then incubated at 37°C for 48 h. Results were evaluated by analyzing the presence or absence of growth in the SDA (Bagiu et al., 2012).

### Activity of the Free Extract in Combination With Clinically Used Antifungals

We determined the *in vitro* activity of the free extract in combination with fluconazole (Pfizer Inc., New York, NY, United States), amphotericin B (Sigma Chemical Co., St. Louis, MO, United States) and caspofungin (Sigma Chemical Co., St. Louis, MO, United States) (as representatives of each class of antifungal drugs, azoles, polyenes and echinocandins) using a checkerboard titration method. A round-bottom 96-well microplate (Corning Inc., Corning, NY, United States) was filled with RPMI medium supplemented with L-glutamine (Cellgro; Corning, United States) and buffered with 165 mM morpholinepropanesulfonic acid (MOPS) (Thermo Fisher Scientific Inc., United States) at pH 7.0 (50 μL) from column 2–10. Drug A (established antifungal) (100 μL) was added in column 1 and serial 2-fold dilutions (50 μL) was performed from column 1–9. In another microplate, 50 μL of RPMI were dispensed from row B to H, and drug B (the free extract) (100 μL) was added to wells in row A (column 1–10) and subsequently diluted 2-fold from row A to G. The different dilutions of drug B were then transferred to the microplate containing dilutions of drug A. In this scheme, row H (columns 1–9) and column 10 (rows A-H) contain drugs A and B alone, respectively. The yeast inoculum (100 μL in RMPI medium) was added from columns 1 to 11 (rows A–H) at a final concentration of 1 × 10^3^ cells/mL, and then microplates were incubated at 37°C for 48 h. Columns 11 and 12 represent growth and media controls. MIC values were evaluated by visual analysis and confirmed by spectrophotometry at 490 nm in a microtiter plate reader (Benchmark Microplate reader; Bio-Rad, CA, United States).

The results were analyzed using the Fractionary Inhibitory Concentration (FIC) indices, a non-parametric model based on the Loewe additivity theory. The FIC indices were defined as the following equation: FICI = (FIC_A_ + FIC_B_), where: FIC_A_ = (MIC_Acombination_/MIC_Aalone_) and FIC_B_ = (MIC_Bcombination_/MIC_Balone_). MIC_Aalone_ and MIC_Balone_ are the Minimum Inhibitory Concentrations (MIC) of drugs A and B alone and MIC_Acombination_ and MIC_Bcombination_ are the concentrations of drugs A and B at the isoeffective combinations, respectively. Off-scale MICs were considered as the lowest or the highest concentrations tested in the microdilution. The FICI was interpreted as ‘synergy’ (FICI ≤ 0.5), ‘indifference’ (FICI > 0.5–4.0) and ‘antagonism’ (FICI > 4.0) (Hindler, 2010).

### Serial Passage Experiments to Evaluate the Potential for the Development of Resistance

Once we had established the activity of our samples, we performed a series of serial passage experiments to evaluate the potential to induce resistance upon repeated exposure to the free extract, similar to previous experiments looking at development of resistance (Pierce et al., 2015; Romo et al., 2018). In these experiments, experimental *C. albicans* or *C. glabrata* populations were exposed to sub inhibitory concentrations of the free extract (at 1/2 the MIC_50_) or fluconazole. Cultures were placed in orbital shakers and every day, 10 μL from each culture was serially transferred into 1 mL of fresh medium containing the sample. These daily transfers were performed for 8 weeks and after the first week, the strains were exposed to the MIC_50_ and this was continued uninterrupted until the end of the experiment (in the case of fluconazole, as increased resistance was detected, the concentrations were doubled every week). After daily transfers, population samples were archived in 1 mL of 40% (vol/vol) glycerol at −80°C. Cells from populations recovered at different time points were then used for the determination of MICs against the free extract and fluconazole using the same 96-well microtiter plate methodology (Clinical and Laboratory Standards Institute, 2008).

### Activity of the Free Extract Against *Candida* Biofilms

The activity of the free extract against *C. albicans* and *C. glabrata* biofilms under two different treatment modalities (inhibition of biofilm formation and activity against preformed biofilms) was evaluated using the 96-well microtiter plate of *Candida* biofilm formation and susceptibility testing (Pierce et al., 2008, 2010). Briefly, for inhibition of biofilm formation the media RPMI 1640 (50 μL) was added to each well in a flat-bottom 96-well microplate (Corning Inc., NY, United States), the free extract (50 μL) was added in column 1 and serially diluted until column 10 at concentrations ranging from 500 to 0.97 μg/mL. Then 50 μL of the yeast suspension at a concentration of 2 × 10^6^ cells/mL were added to well of columns 1–11, and microtiter plates were incubated for 24 h at 37°C. To determine the activity against preformed biofilms, the yeast inoculum (100 μL of a suspension of 1 × 10^6^ cells/mL in RPMI medium) was added to each well of a flat-bottom 96-well microplate and was incubated at 37°C for 24 h in order to allow for biofilm formation. After this period, the liquid was carefully aspirated not to touch the preformed biofilm, which was then washed with PBS (100 μL) two times in order to remove planktonic and non-adherent cells. In another microplate, the dilutions of the free extract were prepared from 1000 to 1.95 μg/mL, and transferred to the microtiter plate containing the preformed biofilms, which was incubated for an additional 24 h at 37°C. After the incubation periods, the liquid in each well of the microplates from both assays was carefully aspirated and the biofilm was washed with PBS (100 μL) twice in order to remove planktonic and non-adherent cells. The post-processing to measure the metabolic activity after the antifungal treatment was evaluated by XTT (Sigma) reduction assay as previously described by our group (Pierce et al., 2008, 2010). Finally, plates were read by spectrophotometry at 490 nm in a microtiter plate reader.

### *In vitro* Cytotoxicity Assays Using Cell Lines

VERO (ATCC^®^ CCL-81^TM^), MRC-5 (ATCC^®^ CCL-171^TM^) and HaCaT cell lines were used to determine cytotoxicity of the free and loaded extracts by determining IC_50_ values (the highest concentration of the sample that allows the viability of at least 50% of the cells) according to Pavan et al. (2010). Cells were incubated at 37°C with 5% of CO_2_ in plates with a surface area of 12.50 cm^2^ in 10 mL of DMEM (Vitrocell^®^, Campinas, SP, Brazil) supplemented with 10% of fetal bovine serum, gentamicin sulfate (50 mg/L) and amphotericin B (2 mg/L). This technique entails collecting the cells using a solution of trypsin/EDTA (Vitrocell^®^), centrifuging the cells at 2,000 rpm for 5 min, counting the number of cells in a Neubauer chamber, determining viability using a 0.4% of Trypan blue solution – 0.25% (Sigma-Aldrich^®^, St. Louis, MO, United States), which is excluded from viable cells, and adjusting the cell concentration to 2.5 × 10^5^ cells/mL in DMEM. Next, 200 μL of the suspension were deposited into each well of a 96-well microplate (KASVI^®^, Curitiba, PR, Brazil) and samples were then incubated at 37°C in an atmosphere of 5% of CO_2_ for 24 h to facilitate the attachment of the cells to the well (O’Brien et al., 2000). Dilutions of the tested samples were prepared to obtain concentrations ranging from 1,000 to 3.90 μg/mL. The dilutions were added to the cells after the medium and the non-adherent cells were removed. Cells were then incubated for an additional 24 h. The cytotoxicity of the samples was determined by adding 30 μL of resazurin and reading after an incubation period of approximately 6 h. The reading was performed using a microplate Spectrafluor Plus (TECAN^®^, Männedorf, Switzerland) reader using excitation and emission filters with wavelengths at 530 and 590 nm, respectively. This reading is based on the resazurin reduction to the highly fluorescent resorufin through cell viability. Non-viable cells fast lose their metabolic capacity in reducing resazurin and are not able to produce a fluorescent signal. Untreated cells (negative control or viable cells) and cells treated with doxorubicin (Sigma-Aldrich, St. Louis, MO, United States) at 100 nmol (positive control or dead cells), DMSO (5%) and microemulsion (without the active ingredient) were also used in each test as controls. The assays were performed three different times.

The selectivity index (SI) of the extract alone or loaded into the microemulsion was obtained by calculating the ratio of IC_50_ value divided by the MIC value for the fungal strain. A higher SI value indicates that the analyzed extract is more active against the yeast and less cytotoxic to the host, with an SI greater than 10 considered to be a promising value for the samples (Tuberculosis Drug Screening Program, 2001).

### Toxicity Assays Using an Alternative *in vivo* Model of *Artemia salina* L.

The toxicity of the samples was also determined using an *Artemia salina* L. model. The amount of 25 mg of brine shrimp eggs (Natal, RN, Brazil) were incubated in a beaker (2000 mL) containing artificial salt water (temperature ranging from 20 to 30°C). The artificial salt water consisted of 23 g NaCl, 11 g of MgCl_2_ 6H_2_O, 4 g of Na_2_SO_4_, 1.3 g of CaCl_2_ H_2_O, and 0.7 g of KCl dissolved in 1000 mL of distilled water. The pH was adjusted to 9.0 using 5 N NaOH to prevent *Artemia* larvae (nauplii) death due to the low pH during the incubation process. After 1 day, *Saccharomyces cerevisiae* was added to the beaker (0.6 g per liter of salt water) to cultivate the nauplii, which were collected for the experiment after 2 days of egg incubation. For the experiment, 120 μL of a suspension containing 10–15 nauplii was added to each well of a 96-well microtiter plate. In another 96-well microplate, samples were serially diluted resulting in concentrations ranging from 500 to 0.97 μg/mL. After 24 h of incubation, microplates were examined under a Lupa binocular microscope (×3.0), and the number of live nauplii in each was counted in order to determine the lethal dose (LD_50_) (Parra et al., 2001). Untreated nauplii (negative control or viable nauplii) and nauplii treated with potassium dichromate at concentrations ranging from 250 to 1.95 μg/mL (positive control or dead nauplii) were also tested as controls. According to Food and Drug Administration (FDA), LD_50_ can be defined as the sample concentration considered to be lethal to 50% of the organisms after being exposed to the sample to be tested. LD_50_ is normally expressed as a time-dependent variation, i.e., 24 h LD_50_. In all toxicity assays, DMSO 2% (Sigma-Aldrich^®^) and the microemulsion without the extract were used as controls. Assays were performed in three independent experiments.

### Activity of the Free and Loaded Extracts in an *in vivo* Model of Vulvovaginal Candidiasis

The anti-*C. albicans* activity of the free and loaded extract was also tested using an *in vivo* model of vulvovaginal candidiasis This experiment was previously approved by the Ethics Committee on the Use of Animals in Research – CEUA of the School of Pharmaceutical Sciences – São Paulo State University (protocol number 28/2015).

Female Wistar rats (*n* = 6 per group; 150–200 g body weight, 8 weeks age) were immunosuppressed with cyclophosphamide (Sigma^®^, 20 mg/Kg, 0.3 mL – one dose) and pseudo-estrus state was induced with estradiol via subcutaneous administration (Sigma^®^, 0.2 mg/mL once a day until the rats reached an appropriate state). Vulvovaginal candidiasis infection was induced using the *C. albicans* strain SC5314. This strain was originally a clinical isolate obtained from a patient with disseminated candidiasis, and is also the strain used in the *C. albicans* genome sequencing project; as such it has been widely used in virulence studies (Gillum et al., 1984; Swindell et al., 2009; Zago et al., 2015). Moreover, it has been documented that this strain is highly filamentous and capable of forming robust biofilms that display high level of resistance to clinically used antifungals (Pierce et al., 2008). These attributes are ideal for the establishment of vaginal infection in the animal model used, since it is well established in the literature that vaginal candidiasis has a biofilm etiology (Harriott et al., 2010). After the infection with *C. albicans* SC5314 at a concentration of 5.0 × 10^7^ cells/mL (intravaginal inoculation – 100 μL) the free and loaded extract (2× MIC) were topically administered to two different groups of animals twice a day (100 μL) during 8 days of treatment. Appropriate positive (infected and treated with antifungal cream consisted of tetracycline −25 mg/g + amphotericin B – 12.5 mg/g) and negative (infected and untreated) controls were also used (see Figure 2 for description of the different control and experimental groups). The efficacy of the treatment was examined by evaluating the fungal load during the course of the infection via determination of colony forming units (CFU) in vaginal lavage fluids (lavage performed with 100 μL of PBS). After discontinuing the treatment, determination of CFUs in vaginal lavages was performed during 7 consecutive days in order to verify if the disease could eventually reemerge. Statistical treatment was performed and significant differences (*p* < 0.05) were analyzed according to two-way ANOVA with post-test Tukey.

## Results and Discussion

### *In vitro* Activity of the Extract Alone or Incorporated Into the Nanostructured System Against *C. albicans* and *C. glabrata* Collection Strains

*C. albicans* and *C. glabrata* represent the two most common causative agents of candidiasis, affecting an expanding population of at-risk patients. In particular, the number of *C. glabrata* infections has increased in the last few decades, which is generally attributed to the decreased susceptibility of this microorganism to azole antifungals (Whaley et al., 2017; Cavalheiro et al., 2018). Clearly, there is an interest in the development of novel therapeutic approaches for candidiasis that may overcome the problem of resistance (Srinivasan et al., 2014), which has led to an increased interest and reevaluation of the properties of natural products used in folk medicine in different parts of the world (Li and Lou, 2018). Our previous experiments indicated that the hydroethanolic extract prepared from leaves of *A. urundeuva* displayed the highest levels of activity against a *C. albicans* collection strain (ATCC 18804) among different types of extracts prepared from different plants from this same *Astronium* species (Bonifácio et al., 2015). Furthermore, the antifungal activity was retained when the extract was incorporated into a nanostructured lipid system (Bonifácio et al., 2015). Other groups have also reported on the antifungal activity of *A. urundeuva* bark and leaves against different species of *Candida* (Oliveira et al., 2017). Thus, in an initial set of experiments we used CLSI techniques to assess the antifungal activity of both the free extract and the extract incorporated in an improved microemulsion formulation against two other collection strains of *C. albicans*, and also expanded these studies to a *C. glabrata* collection strain (Table 1). As seen in the Table, and confirming our previous results, *in vitro* susceptibility testing results demonstrated similar levels of activity for the free and loaded extracts against both strains of *C. albicans*. Interestingly, an even more potent antifungal activity was detected against the *C. glabrata* collection strain used in these initial experiments.

**TABLE 1:** Inhibitory effect of free and loaded extract on *C. albicans* and *C. glabrata* planktonic cells.

**Strain**	***C. albicans***				***C. glabrata***	
		
**Sample**	**ATCC 10231**		**SC5314**		**ATCC 2001**	
			
	**MIC_90_**	**MFC**	**MIC_90_**	**MFC**	**MIC_90_**	**MFC**
Free	15.62	125	31.25	125	0.24	125
Loaded into ME	15.62	15.62	31.25	31.25	0.24	0.24

*Values are in μg/mL. ME, microemulsion; MIC, minimum inhibitory concentration; MFC, minimum fungicidal concentration.*

We also determined the MFCs for the free and the loaded extracts against the same collection strains. As seen in Table 1, a much higher concentration of the free extract was required to exert a fungicidal effect against the *Candida* strains. However, MFC values for the loaded extract against both species of *Candida* were the same as their corresponding MICs, indicating that a fungicidal effect was exerted at low concentrations.

### Activity of the Free Extract Against *C. albicans* and *C. glabrata* Clinical Isolates Exhibiting Different Levels of Resistance Against Conventional Antifungals

As mentioned before, the emergence of resistance has become a significant clinical problem that often limits our ability to successfully treat *Candida* infections, also highlighting the need for the discovery and development of novel antifungal agents, particularly those active against species and strains that are resistant to conventional antifungals (Ngo et al., 2016; Perlin et al., 2017). In *C. albicans*, azole resistance typically develops upon prolonged therapy, whereas *C. glabrata* commonly shows decreased susceptibility to azole derivatives, with echinocandin resistance also being detected with increasing frequency in last decade (Ngo et al., 2016; Perlin et al., 2017).

In the case of *C. albicans* we examined the activity of our free extract against a number of clinical isolates recovered form HIV-infected patients with oropharyngeal candidiasis, including several isolates that are resistant to treatment with conventional antifungals and for which molecular mechanisms of resistance have been previously described (White, 1997; Perea et al., 2001). Additionally, we conducted susceptibility testing assays of the free extract against several laboratory generated strains overexpressing key transcriptional activators of drug resistance efflux pumps and the ergosterol pathway (Schubert et al., 2011; Flowers et al., 2012); including a gain-of-function strain in *UPC2*, leading to overexpression of *ERG11* (the gene encoding lanosterol demethylase, the target enzyme of azole antifungals), as well as gain-of-function strains in *TAC1* and *MRR1*, leading to overexpression of *CDR* and *MDR* genes, respectively, which encode efflux pumps involved in azole resistance (Schubert et al., 2011; Flowers et al., 2012). Table 2 contains information on each of these strains as well as the MIC values obtained for the free extract. As seen in Table 2, although most of the MIC values obtained for the different strains were mostly comparable to those obtained for collection strains. However, we detected elevated MIC values, particularly for strains MRR1R34A, 3731 and 2440, which could potentially indicate that the active component in the extract may be a substrate for the Mdr1 major facilitator also involved in azole resistance. Overall these results seem to confirm the antifungal activity of the free extract against a majority of *C. albicans* strains with decreased susceptibility against conventional antifungals, including both clinical isolates and laboratory-generated strains that show resistance against azoles.

**TABLE 2:** Results (MIC values) of *in vitro* susceptibility testing of the free extract against *C. albicans* clinical isolates displaying different mechanisms of azole resistance and against laboratory generated strains with activating mutantions in key genes involved in azole resistance.

**Strain**	**Description**	**Overexpressed genes**	**Extract**	**Fluconazole**
4639	F449S, T229A (Erg11p substitutions)	*MDR1, CDR1*	7.81	>64^a^
4617	F449S, T229A (Erg11p substitutions)	N/A	3.9	64^a^
6482	D116E, K128T, Y132H, D278N, G464S, P230L (Erg11p substitutions)	N/A	125	>64^b^
2440	V437I (Erg11p substitution)	*MDR1, ERG11*	250	64^a^
3731	F126L, K143R (Erg11p substitutions)	*MDR1*	500	>64^a^
412	K128T (Erg11p substitution)	N/A	62.5	0.5^a^
TW2	Clinical isolate (drug resistance observed)	*MDR1*	62.5	2^c^
TW3	Clinical isolate (drug resistance observed)	*MDR1*	62.5	8^c^
TW17	Clinical isolate (multi drug resistance observed) Point mutation: R467K	*CDR1, MDR1, ERG11*	125	128^c^
*TAC1*R34A	Strain with homozygous activating mutation in *TAC1*	*TAC1, CDR1* and *CDR2*	125	–
*MRR1*R34A	Strain with homozygous activating mutation in *MRR1*	*MRR1* and *MDR1*	>500	3.13^d^
*UPC2*R14A	Strain with homozygous activating mutation in *UPC2*	*UPC2* and *ERG11*	15.62	>256^e^

*Results for fluconazole are from previous publications using the same standardized methodologies and are provided for comparative purposes. Values are in μg/mL. Fluconazole MIC values from previous reports: ^*a*^Perea et al., 2001; ^*b*^Li et al., 2004; ^*c*^White et al., 1997; ^*d*^Schubert et al., 2011; ^*e*^Flowers et al., 2012.*

We then evaluated the activity of the free extract against a series of *C. glabrata* clinical isolates from the FTL collection that are resistant to azoles or echinocandins. Confirming our results using the *C. glabrata* collection strain (Table 3), the free extract showed very potent activity against all *C. glabrata* isolates tested, which was completely unaffected by their resistance patterns to clinically used antifungals. Thus, it would seem that the free extract may represent a promising alternative particularly for the treatment of *C. glabrata* infections, including those that may fail conventional therapy.

**TABLE 3:** The inhibitory effect of the free extract and standard antifungals fluconazole and echinocandin against resistant *C. glabrata* clinical isolates.

**Cell line**	**Free extract**		**Fluconazole**		**Caspofungin**	
			
**Sample**	**MIC_50_**	**MIC_90_**	**MIC_50_**	**MIC_90_**	**MIC_50_**	**MIC_90_**
CgSM1 (azole-S)	0.97	1.95	4–8	8	–	–
CgSM3 (azole-R)	0.97	1.95	62.5–125	125	–	–
DI19-56 (azole-R)	<0.97	1.95–3.90	>128	>128	–	–
DI19-57 (azole-R)	<0.97	1.95–3.90	64	128	–	–
DI19-58 (azole-R)	<0.97	1.95–3.90	>128	>128	–	–
DI19-59 (azole-R)	<0.97	1.95–3.90	>128	>128	–	–
DI19-60 (azole-R)	<0.97	1.95–3.90	128	>128	–	–
DI19-61 (echino-R)	0.97–1.95	3.90–7.81	–	–	4	>4
DI19-62 (echino-R)	0.97–1.95	3.90–7.81	–	–	> 4	>4
DI19-63 (echino-R)	0.97–1.95	1.95	–	–	> 4	>4
DI19-64 (echino-R)	0.48–0.97	0.97	–	–	2–4	4
DI19-65 (echino-R)	0.48–0.97	1.95	–	–	2–4	4

*Values are in μg/mL. azole-S, azole-susceptible strain; azole-R, azole-resistant strain; echino-R, echinocandin-resistant strain.*

### *In vitro* Activity of the Free Extract in Combination With Conventional Antifungal Agents

The antifungal activity detected for the free extract makes it a potential candidate for the development of combinatorial therapies together with currently used antifungal agents. In some cases, plant extracts with biological properties, i.e., antifungal, require such high concentrations to be effective that it could be toxic to healthy cells and cause many side effects. The same situation is also applicable to antifungals already used in clinical therapy, i.e., amphotericin B, that is considered nephrotoxic. Therefore, if these plant extracts could be used combined with antifungals, both at lower concentrations, it could lead to increased activity and reduce their negative side effects. To examine the activity of these combinations, we performed checkerboard assays in which different concentrations of the free extract were used in combination with different dilutions of fluconazole, caspofungin or amphotericin B, both against *C. albicans* and *C. glabrata*. As seen in Supplementary Table S1, after calculation of the respective FICIs, a majority of combinations showed indifference, with the notable exemption of the combinations between the free extract and amphotericin B against *C. albicans*, which resulted in synergism. Overall, these results indicate that the free extract could potentially be used in combination with currently used antifungal agents.

### Serial Passage Experiments to Evaluate the Potential for the Induction of Resistance

**FIGURE 1:**
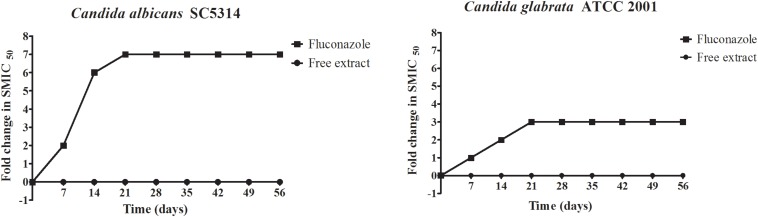
Serial passage of *C. albicans* SC5314 and *C. glabrata* ATCC 2001 in the presence of the free extract or fluconazole to assess the potential for the development of resistance.

*Candida* has the ability to develop resistance upon prolonged and repeated exposure to fluconazole, which greatly complicates therapy (Morschhäuser, 2016; Berkow and Lockhart, 2017). Thus, we were interested in examining if this same conditions could lead to the development of resistance against the free extract, for which we performed serial passage experiments as previously described by our group (Pierce et al., 2015; Romo et al., 2018), both with the free extract and with fluconazole. The initial concentrations used for these experiments were half the MIC values during the first week, which was then increased to the MIC from weeks 2 to 8, at which time the experiment was ended. In the case of fluconazole, as decreased susceptibility was detected after successive passages, the concentrations were doubled each week. As shown in Figure 1, results indicated that *C. albicans* and *C. glabrata* did not develop resistance when exposed to the free extract, as the MIC values were identical at the beginning and end of serial passage experiments. This was in stark contrast to fluconazole which, as expected, led to the induction of resistance in both *Candida* species, with MIC values at the end of experiments being greater than 128 μg/mL.

### Activity of the Free Extract Against Biofilms of *C. albicans* and *C. glabrata*

*Candida* species have the ability to form biofilms on different inert and biological surfaces, and it is well established that biofilm formation leads to high levels of resistance against most antifungal agents (Uppuluri et al., 2009; Rodrigues et al., 2017). Thus, we were interested in examining the activity of the free extract against *C. albicans* and *C. glabrata* biofilms. In a first set of experiments we evaluated the activity of the free extract against preformed biofilms of both *Candida* species, whereas a second set of experiments examined the ability of the free extract to inhibit biofilm formation. As seen in Table 4, preformed biofilms of *C. albicans* and *C. glabrata* were intrinsically resistant to the free extract. However, interestingly the extract was able to inhibit *C. albicans* biofilm formation at concentrations only slightly higher than those inhibiting growth of planktonic cells. The same was not true for *C. glabrata*, as the concentrations of free extract capable of inhibiting biofilm formation were much higher than those effective against planktonic growth. Interestingly, Trentin and co-workers (Trentin et al., 2013) studied stem-bark extracts from different plants, including *A. urundeuva*, and reported on the activity of this extract against *Pseudomonas aeruginosa* biofilms.

**TABLE 4:** Inhibitory effect of the free extract on preformed biofilms and their ability to inhibit biofilm formation of *Candida albicans* and *Candida glabrata*.

**Strain**	**SMIC_50_**	
	
	**Inhibition**	**Preformed**
*C. albicans* SC5314	62.5	1000
*C. glabrata* ATCC 2001	250	1000

*Values are in μg/mL. SMIC, sessile minimum inhibitory concentration.*

### Toxicity Assays

Very often effective antimicrobial concentrations of natural products are relatively high, and their use may be limited due to toxicity (Van Vuuren and Holl, 2017; Wright, 2017). Our previous report pointed to the low cytotoxicity of the free and loaded extracts when tested in an *in vitro* system (Bonifácio et al., 2015). Here, we have expanded upon our initial observations by examining the cytotoxicity of the free extract in three different cells lines, and also using an alternative *in vivo* model in *Artemia salina* L. These experiments were performed to confirm lack of toxicity as a prelude to *in vivo* experiments (see below).

Table 5 shows the IC_50_ cytotoxicity values for the free and loaded extracts when tested in VERO, MRC-5 and HaCaT cell lines. When analyzing the cytotoxicity results of VERO cells line (an epithelial cell from green monkeys), we could observe that the extract loaded into the microemulsion presented a higher IC_50_ (greater than 1000 μg/mL) when compared to the free extract (536.1 μg/mL), and these values were 229 and 123 times greater, respectively, than IC_50_ value observed for doxorubicin (4.36 μg/mL for this cell line), a reference drug used in these assays. Regarding the MRC-5 cell line (a normal cell derived from human lungs widely used for phenotypic screening of drugs), both the free extract and the microemulsion had similar IC_50_ values (360 and 380 μg/mL, respectively), which also compared favorably to doxorubicin (IC_50_ of 1.90 μg/mL). Lower IC_50_ values were detected when the HaCaT (human keratinocytes) cell line was used, indicating a higher toxicity for this cell line compared to the others, although the IC_50_ values for both the free and loaded extracts (158 and 110 μg/mL, respectively) were still significantly higher than IC_50_ values for doxorubicin (6.79 μg/mL). When these IC_50_ values were compared to the corresponding MIC values for both *C. albicans* and *C. glabrata* (see Table 1), the resulting selectivity indices pointed toward a good safety profile associated with these extracts, with the calculated SI values generally higher than 10 in most instances, except for slightly lower values for the tests performed using HaCaT cells.

**TABLE 5:** Biological assessments for the toxicity of the free and loaded extract of *A. urundeuva* leaves in different cell lines as assessed by IC_50_ values of the free and loaded extract in comparison to doxorubicin (used as control).

**Cell line**	**IC_50_ VERO**	**IC_50_ MRC-5**	**IC_50_ HaCaT**
			
**Sample**			
Free extract	536.1	360	158
Loaded extract	>1000	380	110
Doxorubicin	4.36	1.90	6.79

*Values are in μg/mL.*

The toxicity of the free and loaded extracts were further evaluated *in vivo* using a novel alternative model of *Artemia salina* L., which is gaining favor for the evaluation of toxicity properties of a number of natural products (Krishnaraju et al., 2005; Amaral and Silva, 2008; Silva Filho et al., 2009; Nguta et al., 2011; Arcanjo et al., 2012; Mirzaei and Mirzaei, 2013; Lalisan et al., 2014; Rajabi et al., 2015). Our results demonstrated that nauplii viability was unaffected even when exposed to the highest concentration of both the free and loaded extracts tested (500 μg/mL), thereby demonstrating their lack of toxicity in this model. These results are also in agreement with those previously reported by Amaral and Silva (2008), using the same *Artemia salina* L. model to study the toxicity of different plant extracts, including *A. urundeuva*.

### *In vivo* Efficacy of the Free and Loaded Extracts in a Rat Model of Vulvovaginal Candidiasis

We have previously reported on the activity of the free and loaded extracts in a rat model of vulvovaginal candidiasis using a different *C. albicans* strain (ATCC 18804) (Bonifácio et al., 2015). Also, the formulation of the microemulsion system used in this current report was slightly modified by the addition of 0.5% of Poloxamer 407^®^ in order to increase its *in vivo* bioadhesive properties. Thus, we were interested in determining the efficacy of this improved formulation in the same model of vulvovaginal candidiasis, this time also using a different *C. albicans* strain (SC5314), which is the most common strain used in virulence studies (Gillum et al., 1984; Swindell et al., 2009; Zago et al., 2015).

**FIGURE 2:**
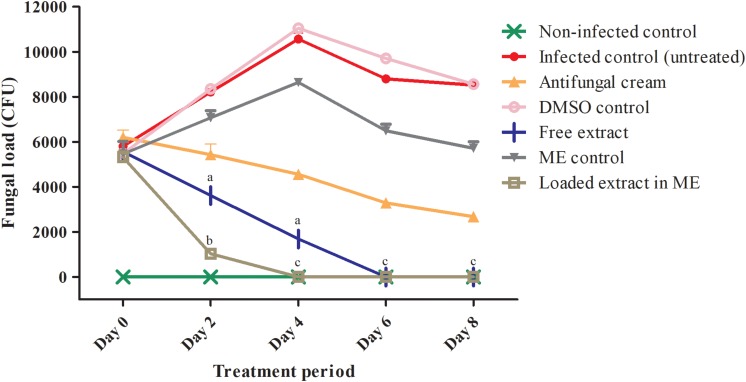
Fungal loads detected in vaginal lavages from animals in different experimental and control groups on days 0, 2, 4, 6, and 8 of treatment. ME, microemulsion. Different letters in superscripts in columns indicate statistically significant differences (*P* < 0.05) according to two-way ANOVA with post-test Tukey. ^a^Comparison between the free extract and the DMSO control. ^b^Comparison between the loaded extract and the microemulsion control. ^c^Comparison between the loaded and the free extract. ^∗^Undetectable fungal burden for the animals treated with the free extract (days 6 and 8) and the loaded extract (days 4, 6, and 8).

As seen in Figure 2, on day 2, presence of CFUs in the lavage fluids recovered from rats in all infected groups confirmed the establishment of vaginal infection, and a trend toward decreased fungal burden was already observed in animals treated topically with either the free or the loaded extract. By day 4 no fungal burden was detected in lavage samples from animals treated with the extract loaded in the improved microemulsion formulation, and the same was true by day 6 in the case of animals treated with the free extract, which compared favorably to the control group of animals treated with a vaginal cream containing amphotericin B which still had significant levels of CFUs. We could also confirm that the control groups treated with vehicle only did not reduce infection, thereby proving that the antifungal activity was truly exhibited by the components from the extract. Interestingly, after the treatment period ended, we could detect a re-infection in the group treated with free extract and the positive control, but not in the case of animals treated with the extract loaded into the microemulsion system (not shown). Thus, together with our previous report (Bonifácio et al., 2015), these results provide further evidence of the *in vivo* antifungal activity of the extract in this model when used topically. Altogether results from this present study demonstrate an improved efficacy of the new microemulsion formulation as compared to our previous report (Bonifácio et al., 2015), with much faster clearance of infection despite the fact that the *C. albicans* strain used in this study forms robust biofilms that exhibit intrinsic resistance to common antifungals, as demonstrated also by the lack of efficacy of our positive control (antifungal cream).

Overall this study furthers our understanding on the antifungal activity of the plant (leaves) extract of *A. urundeuva*, both when used as a free extract and loaded into a microemulsion system. Our results demonstrate that compared to its activity against *C. albicans*, it displays an even more potent activity against *C. glabrata*, and organism that frequently shows decreased susceptibility to conventional antifungals, and preliminary studies indicate that it also displays activity against other *Candida* species, including *C. krusei* and *C. parapsilosis*, as well as against *Cryptococcus neoformans* (not shown). Moreover, the extract is active against a majority of clinical isolates of both *C. albicans* and *C. glabrata* that show resistance against clinically used antifungal agents, and is able to inhibit biofilm formation by both *Candida* species. The extract may be used in combination with conventional antifungals, to improve the efficacy of treatment that even against the clinical isolates considered resistant to most conventional drugs commonly used in treatments. In addition, results from serial passage experiments indicate that the extract is not likely to induce resistance in either *C. albicans* or *C. glabrata*, in stark contrast with results obtained for fluconazole. We have also expanded on the studies on the toxicity properties of the extract, including lack of toxicity *in vivo* in an alternative model of *Artemia salina* L. Our *in vivo* experiments in the rat model of *Candida* vaginitis demonstrate the efficacy of treatment with the free and loaded extract. Altogether, our results confirm the antifungal properties of the *A. urundeuva* leaves extract both *in vitro* and *in vivo*, and its promise to be further developed as an alternative treatment for the treatment of candidiasis and potentially other fungal infections.

## Data Availability Statement

The raw data supporting the conclusions of this manuscript will be made available by the authors, without undue reservation, to any qualified researcher.

## Ethics Statement

The animal study was reviewed and approved by the Ethics Committee on the Use of Animals in Research – CEUA of the School of Pharmaceutical Sciences – São Paulo State University (protocol number 28/2015).

## Author Contributions

BB performed all the *in vitro* and *in vivo* assays and drafted the manuscript. TV contributed to the *in vitro* susceptibility assays. IM and MS contributed to the *in vivo* assay. PS and MC contributed to the microemulsion development. IS, ÉO, and FP contributed to the toxicity assay. LS and WV contributed to the extract obtention process. JL-R contributed to all the *in vitro* susceptibility assays, assisted in drafting, and reviewing the manuscript. TB conceived the project, supervised all the assays, assisted in drafting, and reviewing the manuscript.

## Conflict of Interest

The authors declare that the research was conducted in the absence of any commercial or financial relationships that could be construed as a potential conflict of interest.
