# Supplementary material for: Antifungal Activity of a Hydroethanolic Extract From Astronium urundeuva Leaves Against Candida albicans and Candida glabrata
Source: Front Microbiol. 2019 Nov 15;10:2642. doi: 10.3389/fmicb.2019.02642 (PMC6873212; doi:10.3389/fmicb.2019.02642)
Supplement: Supplementary file 1 [file Table_1.DOC]

**Table S1.** The inhibitory effect of the free extract in combination with amphotericin B, fluconazole and echinocandin against *Candida albicans* SC5314 and *Candida glabrata* ATCC 2001. Numbers in parenthesis indicate FIC index for synergistic combinations.

| **Drug**  **Strain** | **Free extract in combination with** | | |
| --- | --- | --- | --- |
|  | **Amphotericin B** | **Fluconazole** | **Caspofungin** |
| *C. albicans* SC5314 | Synergy (0.28) | Indifference | Indifference |
| *C. glabrata* ATCC 2001 | Indifference | Indifference | Indifference |
